# External Evaluation of a Predictive Model of Suboptimal Cytoreduction in Advanced Ovarian Cancer

**DOI:** 10.3390/diagnostics16040624

**Published:** 2026-02-20

**Authors:** Anna Serra Rubert, Maria Victoria Ibañez Gual, Maria Teresa Climent Martí, Vicente Bebia, Antonio Gil-Moreno, Berta Díaz-Feijóo, Nadia Veiga Canuto, Juan Carlos Muruzábal, Gregorio Lopez-Gonzalez, Álvaro Tejerizo, Antoni Llueca

**Affiliations:** 1 Department of Gynecology and Obstetrics, University General Hospital of Castelló, 12004 Castelló, Spain; climarma@gmail.com (M.T.C.M.); antonillueca@gmail.com (A.L.); 2 Department of Medicine, University Jaume I (UJI), 12006 Castelló, Spain; 3 Department of Mathematics, IMAC (Institut Universitari de Matematiques i Aplicacions de Castelló), University Jaume I (UJI), 12071 Castellon, Spain; mibanez@uji.es; 4 Gynecologic Oncology Unit, Department of Gynecology, Hospital Universitari Vall d’Hebron, Universitat Autònoma de Barcelona, 08035 Barcelona, Spain; vicente.bebia@vallhebron.cat (V.B.); antonio.gil@vallhebron.cat (A.G.-M.); 5 Gynecologic Oncology Unit, Clinic Institute of Gynecology, Obstetrics, and Neonatology, Hospital Clinic of Barcelona, Institut d’Investigacions Biomèdiques August Pi i Sunyer (IDIBAPS), Universitat de Barcelona, 08036 Barcelona, Spain; 6 Department of Gynecologic Oncology, Complejo Hospitalario de Navarra, 31008 Pamplona, Spain; naveigac@gmail.com (N.V.C.);; 7 Gynecologic Oncology Unit, Department of Obstetrics and Gynecology, Hospital Universitario 12 de Octubre, 28041 Madrid, Spain; 8 Instituto de Investigación i+12, Universidad Complutense de Madrid, 28041 Madrid, Spain

**Keywords:** advanced ovarian cancer, peritoneal carcinomatosis, predictive model, primary surgery, suboptimal cytoreduction

## Abstract

**Objective:** The aim of this thesis was to externally validate a predictive model of suboptimal surgery in advanced ovarian cancer, developed by doctors Escrig and Llueca. The model classifies patients pre-surgically to estimate the likelihood of incomplete cytoreductive surgery. **Methods:** A retrospective cohort comparison between two time periods was performed. Validation used a new cohort of 83 patients with advanced ovarian cancer, prospectively collected between 2017 and 2023 across five hospitals (experimental group). This group was compared with the original control cohort (2013–2016), which had served for model development. The predictive models (R3 and R4) are based on the Peritoneal Carcinomatosis Index (PCI) assessed by CT, laparoscopic PCI, and the presence of intestinal sub-obstruction. For model R4, intraoperative PCI was also included. **Results:** The experimental group had a lower rate of suboptimal cytoreduction compared with the control group (4.8% vs. 13.8%; *p* = 0.049). Significant differences were observed in ascites (49.4% vs. 27.5%; *p* = 0.002), and no patient in the experimental group presented intestinal sub-obstruction (0% vs. 8%; *p* = 0.002). Although at least 13 suboptimal surgeries were expected for validation, only four occurred. The predictive models did not classify any of these four cases as high risk, instead categorizing them as low or intermediate risk. **Conclusions:** Statistical external validation could not be performed due to event scarcity. This reduced incidence is attributed to selection bias: highly experienced surgical teams from participating centres likely applied criteria similar to those of the model, referring high risk patients (e.g., with intestinal sub-obstruction) to neoadjuvant therapy and thus avoiding suboptimal primary surgeries. Although direct validation was not possible, the findings indirectly suggest that the model is effective in guiding patient selection and improving surgical outcomes.

## 1. Introduction

Ovarian cancer is the principal cause of death related to gynecological cancer, being responsible for 5% of female cancers [1]. In Spain, approximately 8 per 100,000 women per year develop ovarian cancer [2]. A critical aspect of this pathology is its predominantly advanced stage diagnosis (III and IV), which results in a low five-year survival, below 20–30%. Early-stage disease is asymptomatic, making early detection difficult [3].

The standard treatment for advanced ovarian cancer has remained consistent in recent decades, involving primary cytoreduction followed by platinum-based chemotherapy [4]. A determining factor for the survival of these patients is achieving complete cytoreduction during primary surgery, that is, the resection of all visible tumour tissue. The volume of post-surgical residual disease is the most important prognostic factor for progression-free and overall survival [5]. However, achieving complete cytoreduction is challenging, with rates ranging between 15% and 85% in series on cytoreductive surgery for advanced ovarian cancer [6]. These often-extensive procedures entail high postoperative complication rates (11–67%) and mortality rates of 0 6.7% [7].

Given the importance of predicting the capacity to achieve complete cytoreduction, several predictive models have been described in recent years. These models aim to quantify the contribution of various factors (clinical, analytical, radiological) to predict prognosis or the outcome of an intervention, thereby optimizing clinical decision making and potentially reducing costs without compromising patient safety [8].

In this context, the team of doctors Escrig and Llueca, based on data from cytoreductive surgeries performed between 2013 and 2019 at the General Hospital of Castellón, developed a predictive model of suboptimal cytoreduction [9]. This model, designed to classify patients pre-surgically according to the probability of not being able to perform complete surgery, includes two variants:

- Model R3, which uses predictive information obtained solely from pre-surgical tests (PCI CT, laparoscopic PCI, and presence of intestinal sub-obstruction).
- Model R4, which additionally incorporates the intraoperative PCI.

**Figure 1:**
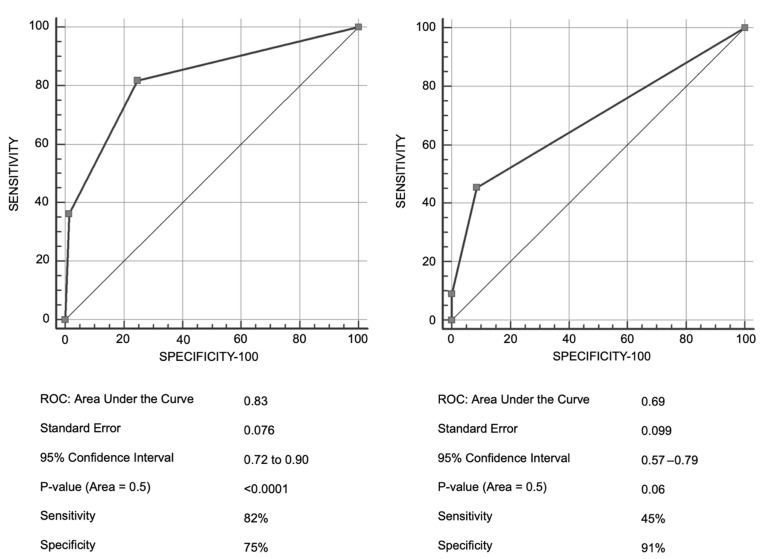
Discrimination between R4 and R3 models.

Initially, model R3 showed a sensitivity of 45% and a specificity of 91%, whereas model R4 showed a sensitivity of 82% and a specificity of 75%, as shown in Figure 1.

The main objective of the present study was to attempt an external validation of this predictive model with prospectively collected data from several collaborating Spanish hospitals, all of them referral centres for this type of surgery. Secondarily, we sought to compare the characteristics of the current surgical population with those of the model’s original population, and to determine whether knowledge derived from this model has influenced patient selection for surgical treatment.

## 2. Materials and Methods

The present study was designed as a retrospective cohort comparison between two time periods for the comparison of populations.

Two cohorts of patients were used:

- Control Group (model design population): Patients diagnosed and treated in the Multidisciplinary Unit of Abdomino Pelvic Oncologic Surgery (MUAPOS) of the University General Hospital of Castellón, between January 2013 and December 2016. This cohort was the basis for the design of the suboptimal surgery model in ovarian cancer [9].
- Experimental Group (validation population): Patients diagnosed with advanced ovarian cancer and treated between January 2017 and March 2023. This cohort was prospectively collected and included the participation of five collaborating hospitals in Spain: Vall d’Hebron Hospital (Barcelona), Clínic Hospital (Barcelona), 12 de Octubre Hospital (Madrid), University Hospital of Navarre (Pamplona), and abdomino pelvic surgical reference units of the Valencian Community.

Patients diagnosed with peritoneal carcinomatosis of ovarian origin in FIGO stages III and IV who underwent cytoreductive surgery were included.

Patients were excluded from the primary surgery group if they presented criteria for irresectability during preoperative evaluation (CT or laparoscopy). Specific irresectability factors mentioned include: lung metastases, liver metastases involving three or more segments, involvement of the hepatic pedicle, and diffuse involvement of the small bowel serosa.

Study data were collected and managed using the REDCap electronic data capture platform, hosted at the Jaume I University. REDCap is a secure web-based tool that facilitates validated data capture for research studies, including audit trails and automated export procedures [10]. Information was anonymized by the participating centres. Variables collected included: age, FIGO stage, PCI CT, laparoscopic PCI, intraoperative PCI, presence of ascites, pleural effusion, intestinal sub-obstruction, number of visceral resections, number of lymph nodes removed, postoperative complications, and in-hospital mortality.

*Interventions and Procedures*

Prior to surgery, all selected patients underwent a preoperative study that included:

- Computed Tomography (CT): Preferably CT enterography, for quantification of the Peritoneal Carcinomatosis Index (PCI). A specific protocol for CT enterography was attached.
- Diagnostic Laparoscopy: Performed to assess disease resectability, quantify the PCI, and obtain samples for diagnostic confirmation. Informed consent was obtained from patients.

Once the diagnosis was confirmed and in the absence of exclusion criteria for surgery, an operation was carried out in the referral hospitals. All surgeries were performed under general anesthesia via laparotomy (xipho–pubic incision). The PCI was also calculated intraoperatively. At the end of the intervention, the cytoreduction index was assessed, classifying it as complete (no residual tumour), optimal (tumour residual ≤1 cm), or suboptimal (tumour residual >1 cm).

*Predictive Model*

The predictive model developed by Llueca et al. is based on the presence/absence of four risk factors: PCI CT > 20, laparoscopic PCI > 20, presence of intestinal sub-obstruction, and intraoperative PCI > 20 [9]. Point allocation for each factor is shown in Table 1.

Scores are summed to obtain a Risk Score. Two models were defined:

- Risk Score R3: Sum of PCI CT, laparoscopic PCI, and intestinal sub-obstruction. Risk prediction: Low (0–1 points), Intermediate (2–3 points), High (4 points).
- Risk Score R4: Sum of PCI CT, laparoscopic PCI, intestinal sub-obstruction, and intraoperative PCI. Risk prediction: Low (0–2 points), Intermediate (3–4 points), High (5–6 points).

*Statistical Analysis*

The first part of the study consisted of a descriptive comparison of both populations. Frequency tables and percentages were used for qualitative variables and means and standard deviations for quantitative variables. Comparisons were made using Student’s *t*-test for means and the chi-square test for distributions of qualitative variables.

For external validation of the predictive model, R3 and R4 scores were calculated for each patient in the new database. Contingency tables were constructed, crossing the risk level assigned by the model with the degree of cytoreduction achieved. The discriminatory capacity of the models was evaluated using ROC (Receiver Operating Characteristic) curves and the Area Under the Curve (AUC). The validity parameters—sensitivity (percentage of patients with suboptimal surgery classified as high risk) and specificity (percentage of patients with complete/optimal surgery classified as medium/low risk)—as well as predictive values, were planned to be calculated from these contingency tables.

Sample size calculation for the new experimental cohort was based on a discrimination objective (AUC) of 75%, with a null hypothesis of 0.5 and a positive/negative case ratio of 5.7 (equivalent to a prevalence of suboptimal surgery of 15% in the control group). A required sample of 119 patients was estimated (16 with suboptimal surgery and 92 with complete/optimal surgery, adding 10% for potential losses).

We will provide our data for independent analysis by a selected group of the Editorial Team for additional data analysis or for the reproducibility of this study in other centres if such is requested.

## 3. Results

### 3.1. Characteristics and Comparison of Populations

This prospective multicenter study included a total of 83 patients in the experimental group, treated between January 2017 and March 2023 across several high-volume tertiary referral centres specializing in advanced ovarian cancer surgery. The distribution of cases was as follows: Vall d’Hebron University Hospital (28.5%), Hospital Clínic of Barcelona (19.3%), 12th of October University Hospital (3.6%), the Abdominopelvic Reference Units of the Valencian Community (38.5%), and the University Hospital of Navarra (12%). The control group comprised 80 patients, selected using the same inclusion criteria. The collected data is shown in Table 2.

When comparing demographic and clinical characteristics between the current population (experimental) and the model design population (control), the following observations were made:

- Age: The mean age showed no significant differences (experimental: 61.30 ± 11.26 years; control: 59.9 ± 10.1 years; *p* = 0.41).
- FIGO Stage: No significant differences in the distribution of FIGO stages (IIIC or IV) between the samples (*p* = 0.39).
- Cytoreduction Achieved: A statistically significant decrease in the rate of suboptimal surgeries was observed in the experimental group (4.8% suboptimal; n = 4) compared with the control group (13.8% suboptimal; n = 11) (*p* = 0.049). The complete/optimal cytoreduction rate in the experimental group was 95.2% (n = 79).
- Categorized PCI CT: No significant differences in distribution of PCI CT categories (1-10, 10-20, >20) (*p* = 0.11).
- Categorized laparoscopic PCI: Although differences were not significant (*p* = 0.26), note that 38.8% of the model sample lacked this value, whereas the information was complete in the current sample.
- Categorized intraoperative PCI: No significant differences (*p* = 0.17), although the *p* value (0.079) for the > 20 category was close to statistical significance, suggesting a trend towards lower PCI > 20 in the current group.
- Number of visceral resections per patient: No significant differences in mean values (*p* = 0.16), although the maximum number of visceral resections was lower in the current group (9 vs. 14).
- Number of lymph nodes: No significant differences in the mean number of nodes removed (*p* = 0.28), attributed to a change in clinical practice after publication of the LION study [11].
- Pleural effusion: No significant differences in the presence of pleural effusion (*p* = 0.79).
- Ascites: The presence of ascites was significantly higher in the experimental sample (49.4%) compared with the control sample (27.5%) (*p* = 0.002).
- Intestinal sub-obstruction: A statistically significant difference in the presence of intestinal sub-obstruction was found, being 0% in the experimental group and 8% in the control group (*p* = 0.002).
- In-hospital death: No significant differences in the percentage of in-hospital deaths (*p* = 0.63).

### 3.2. Model Validation Study

**Figure 2:**
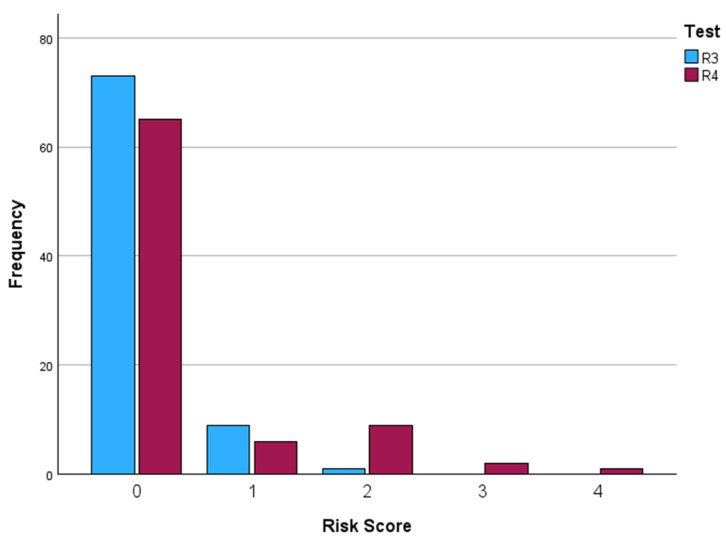
Shows the distribution of R3 and R4 scores in the experimental cohort, illustrating the absence of patients classified as high risk and the strong left-skew of score distribution.

When applying models R3 and R4 to the experimental sample, risk scores were obtained (Figure 2, Table 3).

In model R3, none of the patients obtained a score indicating a high risk of suboptimal surgery. Only one woman reached intermediate risk (2 points); nevertheless, she achieved complete/optimal cytoreduction. The four suboptimal surgeries reported in the experimental group were classified as low risk by model R3 (three with value 0, one with value 1).

Similarly, in model R4, the maximum score obtained was 4 points (intermediate risk). Three of the four patients with suboptimal surgery were classified as low risk by model R4 (two with value 0, one with value 1), and one was classified as intermediate risk (value 3).

The distribution of risk factors in patients with suboptimal surgery in the current cohort was: only one of the four suboptimal patients had a laparoscopic PCI > 20; none had a PCI CT > 20; and one had an intraoperative PCI > 20.

Defining the sensitivity as the proportion of suboptimal surgeries classified as “high risk” and the specificity as the proportion of complete/optimal surgeries classified as “low/intermediate”, sensitivity was 0/4 (0%) and specificity was 79/79 (100%) for both models (R3 and R4). Given the low number of outcome events, exact (Clopper–Pearson) binomial confidence intervals were calculated for sensitivity and specificity to reflect estimation uncertainty (Table 4). These exact intervals show the wide uncertainty driven by the very low number of events, and support interpreting discrimination/performance results as exploratory in this cohort.

## 4. Discussion

Although this external validation study did not allow direct mathematical validation of the predictive model, it provides valuable insight into how the model has influenced clinical practice and patient selection in referral centres. The higher rate of complete and optimal cytoreduction in the experimental group (95.2% vs. 86.3%) suggests improved surgical outcomes in highly experienced centres. This likely reflects stricter patient selection, partly guided by the model. However, intraoperative visual assessment of cytoreduction remains subjective. Emerging biomarkers such as circulating tumour DNA (ctDNA) [12], which correlates with residual tumour burden, may offer a more objective tool for postoperative evaluation.

Ascites was more frequent in the experimental group (49.4% vs. 27.5%), although its direct impact on achieving complete cytoreduction remains unclear. While ascites is associated with advanced disease and poorer prognosis, it does not consistently preclude complete resection [13]. The reason for its higher prevalence in the experimental cohort could not be determined.

A particularly relevant—and paradoxical—finding was the complete absence of intestinal sub-obstruction in the experimental group (0% vs. 8%), a significant difference. As this condition is a strong adverse prognostic factor and contributes two points to the predictive model, the most plausible explanation is that high risk patients were redirected to neoadjuvant therapy. Thus, although the model’s sensitivity for identifying “high risk” cases could not be directly validated, its influence on clinical decision-making indirectly supports its effectiveness in preventing suboptimal surgery.

Accurate prediction tools are essential in advanced ovarian cancer. While the FIGO stage is prognostically relevant, it does not adequately describe tumour burden or distribution. The Peritoneal Carcinomatosis Index (PCI) provides a more precise assessment by quantifying tumour extent across 13 abdominopelvic regions and is generally more informative than other scoring systems, as Eisenkop and Fagotti [14,15]. In this study, CT enterography was preferred for preoperative PCI assessment due to its improved correlation with surgical findings. Despite ongoing exploration of MRI and PET-CT, conventional CT—particularly CT enterography—remains the most practical and cost-effective imaging modality for evaluating peritoneal carcinomatosis in the cytoreductive setting [16,17,18].

The main limitation of this study was the very low number of suboptimal surgeries (four cases), which reduced statistical power. This was further influenced by the COVID-19 pandemic, which led to surgical delays and increased use of neoadjuvant therapy. Even with the planned sample size, the number of events would have been insufficient for robust ROC-based validation, rendering formal statistical discrimination unfeasible.

Notably, no patient in the experimental group was classified as “high risk” by models R3 or R4, suggesting selection bias. It is likely that the model was used prospectively to identify high-risk candidates and redirect them to alternative strategies, such as neoadjuvant chemotherapy. Although this limited conventional statistical validation, it demonstrates the model’s practical value in reducing suboptimal surgeries and associated morbidity.

In summary, while formal mathematical validation was not achievable due to the low event rate and the model’s influence on patient selection, this limitation reflects its integration into clinical practice. The findings suggest that the model contributes to improved patient selection and optimized surgical outcomes in specialized centres.

### 4.1. Strengths and Weakness

This study presents several noteworthy strengths. First, its multicenter and prospective design enhances the generalizability and applicability of findings across different high-volume surgical centres. The collaboration with tertiary referral hospitals specialized in advanced ovarian cancer ensures high surgical expertise and methodological robustness. Furthermore, the rigorous assessment of prognostic variables, including the Peritoneal Carcinomatosis Index (PCI) via computed tomography, laparoscopy, and intraoperative evaluation, provided objective stratification of surgical risk. Notably, although mathematical validation of the predictive model was not achieved, the study indirectly demonstrates the model’s clinical utility, as reflected in the significantly reduced rate of suboptimal cytoreduction (4.8% in the validation cohort versus 13.8% in the original model cohort). This suggests that the model, or knowledge derived from it, has been effectively integrated into clinical decision-making processes in participating centres.

Nonetheless, the study has several limitations. The primary limitation lies in the inability to perform a formal statistical validation due to the low incidence of suboptimal surgeries in the experimental cohort. A clear selection bias was evident, likely resulting from the exclusion of high risk patients—such as those with intestinal sub-obstruction—from primary surgery, potentially due to the implicit application of the model itself. Additionally, the sample size fell short of the initially calculated requirement, and external factors such as the COVID-19 pandemic may have further influenced patient inclusion and treatment decisions. The subjective visual assessment of cytoreduction outcomes, despite being standard practice, also introduces the risk of inter-observer variability.

Additionally, although the Peritoneal Carcinomatosis Index (PCI) was assessed using standardized methods (CT, laparoscopy, and intraoperative evaluation) in high-volume referral centres, inter-observer variability was not formally assessed. Variability in PCI estimation may influence score assignment and, therefore, model reproducibility and external validity. Future prospective validations should incorporate formal inter-observer agreement analyses.

### 4.2. Implications for Practice and Future Research

In conclusion, although direct mathematical validation of the predictive model could not be achieved, the data strongly suggest that the model is already influencing clinical practice by aiding in the preoperative selection of candidates for primary cytoreductive surgery. This indirect success underlines its relevance as a clinical decision-making tool to optimize surgical outcomes and reduce morbidity associated with suboptimal procedures.

Future research should focus on the design of prospective controlled studies that include all eligible patients prior to therapeutic decision-making to mitigate selection bias. Validation of the model in international cohorts with differing healthcare settings and surgical standards would provide broader external validation. Incorporating emerging biomarkers such as circulating tumour DNA (ctDNA) could enhance the objective evaluation of residual tumour burden and further refine risk prediction. Additionally, development of dynamic predictive models using artificial intelligence, integrating clinical, radiological, and molecular data, may offer more robust and personalized tools. Finally, the applicability of the model in neoadjuvant settings should be explored, particularly for selecting patients suitable for interval debulking surgery.

## 5. Conclusions

The predictive model of suboptimal surgery in advanced ovarian cancer developed by MUAPOS could not be mathematically validated directly in this study owing to the small number of suboptimal surgeries and a selection bias in the experimental cohort. The statistically significant differences found in patient characteristics, such as the absence of intestinal sub-obstruction in the experimental group, suggest that knowledge derived from the model was used by the collaborating hospitals to select patients, referring those at high risk of suboptimal cytoreduction to neoadjuvant therapy. Although this precludes formal statistical validation of “high risk”, it represents an indirect success by demonstrating the model’s influence and clinical usefulness in optimizing patient selection for primary surgery in referral centres.

## Figures and Tables

**Table 1:** Final scores by presence or absence of risk factors.

**Predictive Factors**	**Points**
PCI-CT ≤ 20	0
PCI-CT > 20	1
Laparoscopic PCI ≤ 20	0
Laparoscopic PCI > 20	1
Intraoperative PCI ≤ 20	0
Intraoperative PCI > 20	2
No intestinal sub-obstruction	0
Intestinal sub-obstruction	2

**Table 2:** Clinicopathologic characteristics of all patients.

	CONTROL GROUP			EXPERIMENTAL GROUP		
	CCS and OCS (n = 69)	SCS (n = 11)	TOTAL (n = 80)	CCS and OCS (n = 79)	SCS (n = 4)	TOTAL (n = 83)
Age (years ± SD)	60 ± 11	58 ± 10	60 ± 11	60.9 ± 11	68.5 ± 12	61.3 ± 11.3
FIGO stage, n (%)						
IllC	53 (27%)	4 (36%)	57 (71%)	60 (75.9%)	4 (100%)	63 (76.8%)
IV	16 (23%)	7 (64%)	23 (29%)	19 (24.1%)	0	19 (23.2%)
CT-PCI	10 ± 6	15 ± 7	11 ± 7			
Categorized CT-PCI, n (%)						
1–10	44 (64%)	5 (46%)	49 (61%)	41 (51.9%)	1 (25%)	42 (50.6%)
10–20	21 (30%)	3 (27%)	24 (30%)	33 (41.8%)	3 (75%)	36 (43.4.4%)
>20	4 (6%)	3 (27%)	7 (9%)	5 (6.3%)	0	5 (6%)
CT Ascites, n (%)	18 (26%)	4 (36%)	22 (28%)	37 (46.3%)	4 (100%)	41 (49.4%)
Clinical-CT Partial Bowel Obstruction, n (%)	3 (4%)	3 (27%)	6 (8%)	0	0	0
CT Pleural Effusion, n 0%)	10 (14%)	2 (18%)	12 (15%)	9 (11.4%)	2 (50%)	10 (12%)
Laparoscopic PCI, n (%)						
1–10	20 (49%)	0	20 (41%)	35 (44.3%)	1 (25%)	36 (43.2%)
10–20	18 (44%)	5 (62%)	23 (47%)	39 (49.4%)	2 (50%)	41 (49.4%)
>20	3 (7%)	3 (38%)	6 (12%)	5 (6.3%)	1 (25%)	6 (7.2%)
Operative PCI. n ± SD	12 ± 8	23 ± 10	14 ± 9	12 ± 6	15.3 ± 9	12.38 ± 6.45
Categorized Operative PCI, n (%)						
1–10	32 (46%)	2 (18%)	34 (43%)	38 (48.1%)	2 (50%)	36 (43.4%)
10–20	24 (35%)	1 (9%)	25 (31%)	30 (38%)	1 (25%)	41 (49.4%)
>20	13 (19%)	8 (73%)	21 (26%)	11 (13.9%)	1 (25%)	6 (7.2%)
Visceral Resections per patient, n ± SD	3 ± 3	4 ± 4	3 ± 3	3 ± 2	3 ± 2	3 ± 2
All Postoperative Complications, n (%)	38 (55%)	7 (64%)	45 (56%)	42 (53.2%)	3 (75%)	45 (54.2%)
Postoperative 90-day Mortality, n (Yo)	2 (3%)	1 (9%)	3 (3.7%)	1(1.3%)	1 (25%)	2 (2.4%)

**Table 3:** Scores obtained in risk prediction model R3 together with cytoreduction achieved in each case.

Risk Prediction (Risk Score R3)	CC Complete/Optimal	CC Suboptimal
LOW (0–1)	78	4
INTERMEDIATE (2–3)	1	0
HIGH (4)	0	0

**Table 4:** Performance metrics of models R3 and R4 with exact (Clopper–Pearson) 95% confidence intervals.

Model	Sensitivity	Specificity	Suboptimal Surgery Rate
R3	0/4 (0.0%; 95% CI: 0.0–60.2)	79/79 (100.0%; 95% CI: 95.4–100.0)	4/83 (4.8%; 95% CI: 1.3–11.9)
R4	0/4 (0.0%; 95% CI: 0.0–60.2)	79/79 (100.0%; 95% CI: 95.4–100.0)	4/83 (4.8%; 95% CI: 1.3–11.9)

## Data Availability

The data presented in this study are available on request from the corresponding author. The data are not publicly available due to privacy restrictions.

## Abbreviations

The following abbreviations are used in this manuscript:

ctDNA	circulating tumour DNA
PCI	Peritoneal Carcinomatosis Index
CT	Computed Tomography
FIGO	International Federation of Gynecology and Obstetrics
CCS	Complete Citorreductive Surgery
OCS	Optimal Citorreductive Surgery
SCS	Suboptimal Citorreductive Surgery
